# Agressions sexuelles à Port-Gentil

**DOI:** 10.11604/pamj.2013.15.152.1884

**Published:** 2013-08-28

**Authors:** Mohamed Maniboliot Soumah, Gladys Rita Olendo, Mor Ndiaye, Mamadou Lamine Sow

**Affiliations:** 1 Service de médecine légale et médecine du travail, Université Cheikh Anta Diop, Dakar, Sénégal; 2 Médecin légiste expert du dommage corporel, médecin du travail, Cabinet du Littoral, Port-Gentil, Gabon

**Keywords:** Agression sexuelle, viol, victime, Gabon, sexual assault, rape, victim, Gabon

## Abstract

Les agressions sexuelles portent atteinte à l'intégrité physique et psychologique des personnes qui en sont victimes et entraînent des conséquences néfastes. Le viol constitue la forme la plus accomplie de ces agressions. Cette étude rétrospective visait à répertorier les cas de violences sexuelles enregistrés auprès des greffes du tribunal et de la cour criminelle de Port-Gentil, et d'en donner une approche épidémiologique et criminologique. La prévalence des agressions sexuelles judiciarisées à Port-Gentil est de 15 cas par an. Parmi ces 45 dossiers d'agression sexuelle, 23 dossiers concernaient un attentat à la pudeur (51,1%) dont 18 cas impliquaient un mineur de moins de 15 ans (78,2% des attentats à la pudeur). Six dossiers concernaient une tentative de viol (13,3%). Onze dossiers concernaient un viol sur une femme de plus de 15 ans (24,4%). Deux dossiers concernaient un outrage public à la pudeur (4,4%) et 3 un détournement de mineur (6,6%). Les victimes étaient âgées de 4 à 65 ans avec un âge moyen de 13,4 ans. La plupart des victimes provenaient d'un milieu social défavorisé, prolétaire (61,1%). Les agresseurs étaient âgés de 16 à 62 ans avec un âge moyen de 29,6. Il importe de se pencher sur ce phénomène pour comprendre les enjeux que représentent les agressions sexuelles. Il faudra prendre en considération la dénonciation de l'agression sexuelle, le traumatisme subi et la prévention de tels crimes.

## Introduction

Les agressions sexuelles représentent un problème social important dont les principales victimes sont des femmes et des enfants. On regroupe sous le terme “agressions sexuelles”, des infractions de gravités différentes dont le viol, l'exhibition sexuelle et le harcèlement sexuel. L'objectif général de ce travail était de répertorier les cas de violences sexuelles enregistrés auprès des greffes du tribunal et de la cour criminelle de Port-Gentil, et d'en donner une approche épidémiologique et criminologique. Les objectifs spécifiques étaient de donner la prévalence des agressions sexuelles à Port-Gentil et de déterminer la nature de ces agressions. Il s'agit aussi d'analyser le profil des victimes et le profil des agresseurs, de décrire les principales étapes du parcours juridique, de définir le taux d'agressions ayant abouti à une condamnation. Aussi, nous devrons démontrer l'existence d'une corrélation entre la décision de justice et la conclusion du certificat médical, et enfin proposer les grandes lignes de prévention, qui permettront de réduire le nombre d'agressions sexuelles.

## Méthodes

Cette étude se déroule à Port-Gentil au Gabon, en Afrique centrale. Le Gabon est limité au Nord par le Cameroun, au Nord-ouest par la Guinée équatoriale, à l'Est par le Congo, à l'Ouest par l'Océan Atlantique. La capitale est Libreville. Le Gabon est divisé en neuf provinces. Port-Gentil, chef-lieu de la province de l'Ogooué maritime, est l'une des principales villes du Gabon. C'est la capitale économique du Gabon car elle est le chef-lieu de la province productrice de pétrole. Le dernier recensement général de juillet 1993, estimait la population à 1 014 976 habitants soit une densité de 3,8 habitants au km^2^, dont 41% de moins de 15 ans et 46% entre 15 et 49 ans, avec un taux de croissance moyenne de 2,4% par an.

Il s'agit d'une étude rétrospective portant sur les dossiers en rapport avec une agression sexuelle, au tribunal correctionnel et à la cour criminelle de Port-Gentil, du 1er janvier 2003 au 31 décembre 2006. Un questionnaire a été remis aux différents greffes concernés. Les items portaient sur les données générales sur l'activité propre à chaque greffe (nombre total d'affaires instruites, nombre total d'affaires jugées, nombre de dossiers instruits pour agressions sexuelles, nombre de dossiers jugés pour agressions sexuelles), les données sur la victime (âge, sexe, profession, celle des parents si mineur, circonstances de l'agression, comportement de la victime), les données sur l'agresseur (âge, sexe, profession, celle des parents si mineur, coexistence d'autres délits, expertise psychiatrique), le parcours judiciaire, les éléments du certificat médical, la nature du délit, les conclusions pénales.

Le questionnaire a été remis à chaque greffier au cours d'un entretien, après explication des objectifs de l’étude. Puis, un autre rendez-vous a été pris pour la collecte des données. Les personnes contactées ont été les greffiers du juge d'instruction, le greffier du greffe correctionnel du tribunal de grande instance, le greffier de la cour criminelle et le greffier en chef. Seuls les dossiers ayant eu une décision de justice ont été retenu. Ils devaient contenir au moins pièces suivantes: un procès-verbal d'instruction, un certificat médical pour les cas de viol, les coups et blessures volontaires, les attentats à la pudeur et les tentatives de viol sur mineur et un jugement du tribunal correctionnel ou un arrêt de la cour criminelle. Les dossiers en instruction ont été exclus ainsi que les dossiers classés sans suites pour défaut de preuves. Le logiciel informatique EPIINFO a été utilisé pour l'analyse statistique de données recueillies. Une étude descriptive a été faite par l'analyse des proportions et des moyennes. Des tests ont été retenus pour réaliser les statistiques: le test de chi2 pour les données qualitatives et la comparaison des proportions, le test de Fisher exact pour les nombres très inférieurs à 5, le test de Yates pour les nombres inférieurs à 5 et le test de Student pour les données quantitatives et la comparaison des moyennes.

## Résultats

### Prévalence

Au niveau du greffe correctionnel, la prévalence des agressions sexuelles judiciarisées à Port-Gentil est de 15 cas par an. Sur 1586 affaires jugées par le tribunal correctionnel durant cette période, 45 dossiers concernaient une agression sexuelle soit une fréquence de 2,8%. Sur 51 affaires jugées par la cour criminelle, 29 dossiers concernaient une agression sexuelle soit une fréquence de 56%. Notre série comportait ainsi 74 cas d'agression sexuelle.

### Nature du délit

Parmi les 45 dossiers d'agression sexuelle du greffe correctionnel, 23 dossiers concernaient un attentat à la pudeur dont 18 cas impliquaient un mineur de moins de 15 ans, 6 dossiers concernaient une tentative de viol, 11 cas de viol sur une femme de plus de 15 ans, 2 cas d'outrage public à la pudeur et 3 cas de détournement de mineur.

Sur les 29 affaires jugées par la cour criminelle, 26 concernaient un viol sur mineur de moins de 15 ans, 2 cas de tentative de viol sur mineur de moins de 15 ans et un cas de viol avec acte de barbarie sur une femme adulte.

Dans notre série, près de la moitié des cas sont des viols (Tableau 1). Les attentats à la pudeur représentaient quant à eux un tiers des cas d'agression sexuelle. La disqualification du viol faute de preuve, permettait de retenir les autres incriminations. Les viols incestueux représentaient 2% des agressions sexuelles dans notre série.

**Tableau 1:** Répartition délits selon le code de procédure pénale gabonais

Nature du délit	N	%
Exhibition sexuelle ou outrage publique à la pudeur	2	2,7
Attentat à la pudeur avec violences sur mineur	5	6,7
Attentat à la pudeur avec violence sur adulte	5	6,7
Attentat à la pudeur sans violence sur mineur	13	17,5
Attentat à la pudeur sans violence sur adulte	00	00
Tentative de viol sur mineur de moins de 15 ans	2	2,7
Tentative de viol sur femme de plus de 15 ans	6	8,1
Détournement et séquestration de mineur	3	4
Viol sur mineur de moins de 15 ans	26	35,1
Viol sur femme de plus de 15 ans	11	14,8
Viol sur une victime de sexe masculin (attentat à la pudeur)	1	1,3
**TOTAL**	**74**	**100**

### Les conclusions pénales

Dans 12 cas, l'agresseur a été jugé non coupable au bénéfice du doute. Dans 83,7% des cas, l'agresseur a été condamné (Tableau 2). La durée des peines variait de 6 mois à 20 ans d'emprisonnement avec une moyenne de 5 ans.

**Tableau 2:** Répartition des peines selon la nature du délit

Nature du délit / Peine	Relaxe	< 2 ans	2 à 5 ans	6-10 ans	> 10 ans	Total
Exhibition sexuelle ou outrage public à la pudeur	1	1				2
Attentat à la pudeur avec violence sur mineur		1	2	2		5
Attentat à la pudeur avec violence sur adulte	1	3		1		5
Attentat à la pudeur sans violence sur mineur	3	8	1	1		13
Tentative de viol sur mineur de moins de 15 ans		1			1	2
Tentative de viol sur femme de plus de 15 ans	2	4				6
Détournement et séquestration de mineur	1	1	1			3
Viol sur mineur de moins de 15 ans	2	4	12	2	7	27
Viol sur femme de plus de 15 ans	2	2	2	4		10
Viol sur victime de sexe masculin		1				1
**TOTAL**	12	26	18	10	8	74

### La victime

Les victimes étaient âgées de 4 à 65 ans avec un âge moyen de 13,4 ans. La tranche d’âge la plus exposée était celle de 11 à 15 ans (66%). Soixante-douze victimes étaient de sexe féminin. Deux victimes étaient de sexe masculin, soit un ratio de 36 en faveur des femmes.

### Circonstances de l'agression

La plupart des victimes provenaient d'un milieu social défavorisé, prolétaire (61,1%). L'absence d'un des parents (famille monoparentale) ou des deux parents (le tuteur est un autre membre de la famille: grand parent, oncle ou tante) est une donnée quasi constante lorsqu'il s'agit de victime mineure. Les agressions sont fréquentes en zone urbaine. Trois agressions ont été commises en zone rurale (4%). L'agression était souvent commise de nuit entre 18H et 7H du matin dans 67,5% des cas et 32.4% des agressions ont été commises de jour, en l'absence des parents ou des tuteurs. Près de la moitié des agressions, dans 40% des cas, se sont déroulées chez l'agresseur. D'autres, se sont produites chez la victime (29,1%) ou dans la rue (18,2%). Aussi 12,7% des agressions ont été commises en brousse, dans des bureaux abandonnés ou derrière le domicile de la victime. Dans notre série, 10,7% des agressions se sont faites en réunion de deux à quatre agresseurs; il s'agissait toujours de viols. Dans 89,3% des cas, l'agression était commise par un seul agresseur. Dans 9 cas, la victime a été agressée plus d'une fois. Il s'agissait toujours d'une récidive par le même agresseur. Dans 60,8% des cas, l'agression a été accompagnée de violence physique dont la strangulation (8,2%) et les coups et blessures volontaires (41,9%). Dans 14% des cas, l'agresseur a usé d'une arme blanche (couteau). Dans 53,5% des cas, la victime a subi des pressions psychologiques et du chantage de la part de l'agresseur. Dans les cas d'agressions sexuelles sur mineur, l'agresseur adulte a appâté le mineur avec de l'argent ou des cadeaux, pour l'attirer chez lui. Dans tous les cas retrouvés, la victime était consciente. Nous n'avons pas retrouvé de cas de soumission médicamenteuse.

### Comportement de la victime

#### L'agresseur

Les agresseurs étaient âgés de 16 à 62 ans avec un âge moyen de 29,6. Les tranches d’âge les plus incriminées étaient celles de 16 à 35 ans (76,5%). Tous les agresseurs étaient de sexe masculin. La majorité des agresseurs viennent d'un milieu social défavorisé; 46,2% des agresseurs sont sans emploi ou en quête d'un emploi. Les employés de société représentent 15% des agresseurs, les chauffeurs de taxi 9,3% des agresseurs. Nous relevions 5,7% d'agresseurs ayant une activité rurale, 3,8% d’élèves, 1,9% d'ingénieurs et 1,9% d'enseignants. L'addiction a été retrouvée dans 7 cas (9,46%). Il s'agissait principalement de consommation d'alcool, ou de chanvre indien.

#### Lien avec la victime

**Figure 1:**
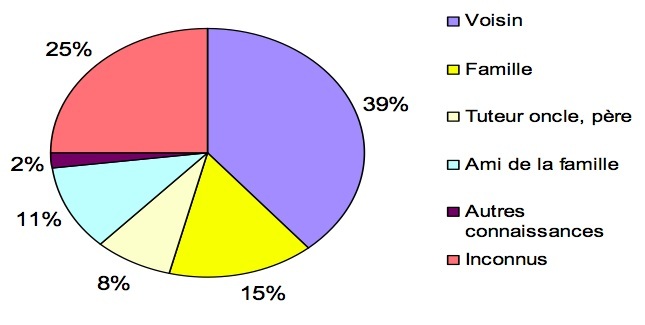
Répartition des agresseurs selon le lien avec la victime

Dans 75% des cas, l'agresseur était connu de la victime. Il s'agissait d'un membre de la famille dans 23% des cas (oncle, cousin, neveu, père), dans 11% des cas d'un ami de la famille, dans 39% des cas d'un voisin, ou d'autres connaissances comme les employés de maison. Dans 25% des cas, il s'agissait d'un inconnu (Figure 1).

### Coexistence d'autres délits

Dans deux agressions, l'agresseur avait commis d'autres délits dans le passé (vol, fraude). Pour neuf agressions, on retrouvait d'autres délits associés comme le défaut de carte de séjour, l'usage et la vente de stupéfiant, les coups et blessures volontaires, la séquestration de mineur, les injures publiques et voies de fait. L'agression sexuelle constituait le seul délit dans 87,8% cas.

### Expertise psychiatrique

Aucune expertise psychiatrique n'a été demandée. Les accusés ont été jugés saints d'esprit et conscient de leurs actes au moment des faits.

### Le parcours judiciaire

Pour toutes les agressions, la plainte avait été déposée dans un commissariat de police avant d’être transmise au parquet puis au juge d'instruction. Le certificat médical est une pièce essentielle du dossier d'instruction en cas de viol ou de coups et blessures volontaires, ou encore en cas d'attentats à la pudeur chez des mineurs. Dans 26,6% des cas, la consultation médicolégale était faite sur réquisition. La consultation médicolégale était faite à l'initiative des victimes dans 35% des cas. La police judiciaire avait orienté les victimes vers la consultation médicolégale dans 38,3% des cas.

### Les éléments du certificat médical

**La qualité du médecin qui délivre le certificat médical:** Dans 73,8% des cas, le médecin était un gynécologue obstétricien. Dans les autres cas, il s'agissait d'un chirurgien (9,5%) ou du médecin chef d'un centre médical périphérique ou rural (14,3%). Dans tous les cas, la consultation médicolégale était faite dans une structure sanitaire publique.

**Le délai entre l'agression et la consultation médicolégale:** variait de 0 à 270 jours avec une moyenne de 24 jours. Un tiers des victimes consultait dans les premières 24 heures.

**Qui accompagne la victime?** Dans tous les cas, la victime mineure était accompagnée d'un des parents.

**Concernant les renseignements cliniques**, l’état général n’était pas évoqué dans 75% des cas. Seuls 7,4% des dossiers portaient des indications sur l’état psychologique de la victime.

**Il n’était pas fait mention de lésions de défense dans les certificats médicaux** Dans tous les cas de suspicion de viol, d'attentat à la pudeur ou de tentative de viol sur mineur, un examen génital avait été fait.

Une incapacité temporaire totale (ITT) était mentionnée dans 14 cas, lorsque la victime présentait des lésions de coups et blessures volontaires comme les morsures de l'agresseur, les coups portés par l'agresseur sur la victime et ayant entraîné un traumatisme du genou, les lésions de strangulation, une grossesse de trois mois. L'ITT variait de 3 à 45 jours avec une durée moyenne de 15 jours. Aucune incapacité permanente partielle (IPP) n’était mentionnée.

Dans 17 cas, une recherche d'infection sexuellement transmissible (IST) avait été proposée aux victimes. Aucun résultat n’était consigné dans les dossiers. Les bilans IST proposés étaient la sérologie VIH (3 cas), sérologie de l'hépatite B (2 cas), recherche de chlamydiae (1 cas), recherche de syphilis (3 cas), prélèvement vaginal (10 cas), prélèvement oral (1 cas). La sérologie de l'hépatite C n’était jamais demandée dans notre série.

Le risque de grossesse était évoqué dans deux cas mais n’était pas recherché. Un cas de grossesse avec un terme de trois mois était diagnostiqué après un viol sur mineur.

La prévention des IST était proposée dans un cas, il s'agissait de prévention des IST d'origine bactérienne avec prescription d'antibiotiques. Aucune prévention des IST virales n’était proposée. Dans deux cas, une infection en relation avec l'agression sexuelle était retrouvée et traitée. Dans un cas, une perforation vaginale était à l'origine d'une péritonite.

## Discussion

Les commissariats de police sont les premiers contacts des victimes avec la justice. Des affaires sont classées sans suites par des arrangements à l'amiable entre famille de victime et d'agresseur, par des médiations privées. Ensuite, de nombreuses agressions sexuelles ne sont pas dénoncées soit par ignorance, soit par rétraction des victimes et de leur famille qui éprouvent un sentiment de honte.

S'agissant de la prévalence, chiffres similaires sont retrouvés au Sénégal [1, 2] avec respectivement 79 cas en 5 ans et 55 cas en en 3 ans; au Cameroun Menick [3] trouvait 224 cas en 6 ans. En Tunisie les chiffres augmentent [4] avec 629 affaires en 6 ans avec une incidence de 14,7 victimes pour 100 000 habitants. La même augmentation est notée dans les séries européennes et américaines [5–7]. Cette différence de chiffres est liée à la constitution de la famille africaine qui limite la judiciarisation des agressions sexuelles; celles-ci sont d'abord réglées entre familles avec une faible autonomie de la volonté de la victime dans une société conservatrice [8].

Dans notre série, près de la moitié des cas sont des viols. Les attentats à la pudeur représentent quant à eux un tiers des cas d'agression sexuelle. Le viol est prépondérant dans toutes les séries [1, 4, 5]. La disqualification du viol faute de preuve, permet de retenir les autres incriminations. Les viols incestueux représentent 2% des agressions sexuelles dans notre série comme au Cameroun [3].

La durée des peines variait de 6 mois à 20 ans d'emprisonnement avec une moyenne de 5 ans. Ces données concordent avec les résultats trouvés au Sénégal [2].

Les victimes sont majoritairement des filles, mineures entre 11 et 15 ans avec une moyenne de 14 ans dans toutes les séries [1, 2, 5, 9] et même 9 ans pour Ménick et entre 3 et 17 ans pour Cécil. Les agressions sur les garçons ne sont pas rares et la série de Gaddour en Tunisie est remarquable par la proportion de 42% des victimes masculines.

Nous n'avons pas retrouvé de cas de soumission médicamenteuse. La difficulté de leur recherche et la prise en charge non spécifique laissent un doute sur l'absence de ces soumissions en milieu africain. Mc Grégor et Coll. évaluaient le risque relatif d'agression sexuelle facilitée par la drogue à 10,7 pour 100 000 [11]. Les produits les plus incriminés sont en France le zolpidem, la zopiclone, le bromazépame, le clonazépam et l'alprazolam [12].

Tous les agresseurs étaient de sexe masculin. Cela est conforme aux données de la littérature [1–3, 7, 8]. S'agissant des agresseurs adolescents, l’étude de Pham TH et al. [13] montre que les adolescents agresseurs ont davantage connu la perte de leur père. Ils présentent davantage de caractéristiques de personnalité soumise, conformiste et d′inhibition sexuelle. Il ne faut pas occulter les cas d'agression par les femmes comme décrits par Denov M. au Canada [14], où les victimes étaient abusées surtout par leur mère, avec un âge moyen de début évalué à cinq ans et une durée moyenne de poursuite des agressions de six ans. L'addiction, chez l'agresseur, a été retrouvée dans 7 cas (9,46%). Il s'agissait principalement de consommation d'alcool, ou de chanvre indien. Perez-Diaz C. trouvait en France des prévenus appartenant à des classes populaires, avec un passé de violence, des antécédents de santé, des problèmes d'alcool et secondairement de santé mentale [15]. Dans 75% des cas, l'agresseur était connu de la victime. Il s'agissait d'un membre de la famille, d'un ami de la famille, d'un voisin, ou d'autres connaissances. Les auteurs faisaient le même constat tant en Afrique que dans le reste du monde [1, 3, 4, 9]. L’école et le milieu sportif, lieux d'apprentissage, sont aussi le lieu de commission des agressions sexuelles [16, 17].

Dans la prise en charge médico-judiciaire, notre étude a permis de montrer que les certificats ne donnaient pas les renseignements attendus dans un certificat délivré à l'issue d'une consultation médicolégale [18]. Les lésions décrites n’étaient pas précises. Mieux les descriptions ne donnaient pas la nature exacte des lésions, leur topographie exacte et leur interprétation médicolégale. La déchirure complète de l'hymen entre 5 heures et 7 heures est communément retenue comme signe de pénétration complète. Les délais longs de consultation rendent certes difficiles les conclusions mais l’étude faite par Mc Cann et Coll. permet de retenir des conclusions malgré la cicatrisation [7]. La tache est plus ardue en cas de lésion anale à distance, la cicatrisation étant meilleure à ce niveau avec le plus souvent disparition des lésions [19]. Les renseignements sur l’état général, l’état psychologique, l'examen somatique n’étaient jamais donnés. L'examen génital était fait dans tous les cas et mentionnait l’état de l'hymen. L'examen anal n’était fait qu'en cas de suspicion de pénétration anale.

## Conclusion

Nous avons vu l'importance des chiffres de la violence sexuelle. Certaines couches de la population sont plus exposées, aucun pays n'en est épargné quel que soit le contexte socioculturel. Il faudra prendre en considération la dénonciation de l'agression sexuelle, le traumatisme subi et la prévention de tels crimes. La violence sexuelle entraîne un mal être, une souffrance muette des hommes et des femmes qui ont en fait les frais. Cette souffrance est autant présente chez la victime que chez l'agresseur. Cette violence peut être entretenue par l'environnement socioculturel et économique. C'est pour ces raisons que nous ne devons pas être uniquement répressifs mais que nous devons prendre aussi des mesures de prévention primaire visant à améliorer la qualité de vie au quotidien des personnes.

## References

[1] Faye Dieme ME, Traore AL, Gueye SM, Moreira PM, Diouf A, Moreau JC (2008). Profil épidémioclinique et prise en charge des victimes d'abus sexuels à la clinique gynécologique et obstétricale du CHU de Dakar. J Gynecol Obstet Biol Reprod..

[2] Soumah MM, Bah H, Mbaye I, Fall MC, Yetognon C, Sow ML (2005). Sévices sexuels sur enfants: corrélation entre les conclusions du certificat médical et les peines retenues. Dakar Med..

[3] Menick DM (2000). Judiciarisation des offenses à caractère sexuel sur mineurs au Cameroun. Méd Trop..

[4] Gaddour N, Mechri A, Lahbib S, Gaha L (2003). Profil épidémiologique et criminologique des abus sexuels dans une région du centre-est tunisien. J Méd Lég Droit Méd..

[5] Burkhardt S, Palmiere C, La Harpe R (2005). Les agressions sexuelles à Genève entre 1999 et 2003. J Méd Lég Droit Méd..

[6] Ingram DM, Everett VD, Ingram DL (2001). The relationship between the transverse hymenal orifice diameter by the separation technique and other possible markers of sexual abuse. Child Abuse Negl..

[7] McCann J, Miyamoto S, Boyle C, Rogers K (2007). Healing of hymenal injuries in prepubertal and adolescent girls: a descriptive study. Pediatrics.

[8] Faye L, Ba I, Seck S, Thiam MH (2010). Particularité clinique et thérapeutique d'un traumatisme psychique: étude d'un cas de viol chez une sénégalaise. Revue Francophone du Stress et du Trauma..

[9] Bah H, Balde S, Dansa K, Soumah M, Telmon N (2006). Agression sexuelle sur mineur en milieu africain: étude de 100 cas au service de médecine légale de Conakry (Guinée). J Méd Lég Droit Méd..

[10] Cecil H, Matson SC (2001). Psychological functioning and family discord among African-American adolescent females with and without a history of childhood sexual abused. Child Abuse Negl..

[11] McGregor Margaret J, Ericksen J, Ronald Lisa A, Janssen Patricia A, Van Vliet A, Schulzer M (2004). Rising incidence of hospital-reported drug-facilitated sexual assault in a large urban community in Canada: Retrospective population-based study. Can J Public Health..

[12] Kintz P (2006). Soumission chimique: à la recherche de l'indétectable. Spectra Biol..

[13] Pham TH, Ducro C, Lemasson A-C (2010). Adolescents auteurs d'agressions sexuelles: aspects étiologiques, diagnostiques et pronostiques. Ann Med Psychol..

[14] Denov Myriam S (2003). To a safer place? Victims of sexual abuse by females and their disclosures to professionals. Child Abuse Negl..

[15] Pérez-Diaz C, Huré MS (2006). Violences, alcool et santé mentale. Alcoologie et Addictologie..

[16] Jolly A, Decamps G (2006). Les agressions sexuelles en milieu sportif: une enquête exploratoire. Science & Motricité..

[17] Nhundu Tichatonga J, Shumba A (2001). The nature and frequency of reported cases of teacher perpetrated child sexual abuse in rural primary schools in Zimbabwe. Child Abuse Negl..

[18] Grill S, Blanc A, Dedouit F, Rougé D, Telmon N (2006). Evaluation de la qualité de rédaction de certificats descriptifs de constatation de coups et blessures volontaires au sein d'une Unité Médico-Judiciaire. J Méd Lég Droit Méd..

[19] Heppenstall-Heger A, McConnell G, Ticson L, Guerra L (2003). Healing Patterns in Anogenital Injuries: A Longitudinal Study of Injuries Associated With Sexual Abuse, Accidental Injuries, or Genital Surgery in the Preadolescent Child. Pediatrics..

